# Postauricular Melanocytic Neuroectodermal Tumor of Infancy: A Rare Site of a Rare Tumor—MNTI as a Postauricular Mass with Literature Review

**DOI:** 10.1155/2018/9829856

**Published:** 2018-07-05

**Authors:** S. Evans, A. Woolley

**Affiliations:** ^1^Department of Otolaryngology Head and Neck Surgery, University of Alabama at Birmingham, Birmingham, AL, USA; ^2^Pediatric Ear, Nose and Throat Associates of Alabama, Children's Hospital of Alabama, Birmingham, AL, USA

## Abstract

Melanocytic neuroectodermal tumor of infancy (MNTI) is a rare osteolytic neoplasm of neural crest cell origin. There are less than 500 documented cases, most frequently affecting the maxilla of infants less than 1 year old. We present a unique case of a two-month-old male with a progressive postauricular mass since birth, confirmed to be a MNTI. The lesion required three resections over the course of five months, with rapid recurrence ultimately requiring a craniectomy, highlighting the difficulty in treating these tumors. Histological and radiographic features were reviewed; an updated literature review for identifying and treating these lesions is presented.

## 1. Introduction

Melanocytic neuroectodermal tumor of infancy (MNTI) is a rare osteolytic neoplasm of neural crest cell origin most frequently found in infants less than 1 year old [1]. Since its original description in 1918 by Dr. Krompecher, less than 500 total cases have been reported in the literature [2]. The most common location for these tumors to present is in the maxilla with approximately two-thirds of lesions occurring there. While typically considered a benign lesion, a malignant potential has been demonstrated at 6.5% and treatment is primarily surgical, though recent advances in tumor genetics have identified mutations conferring chemotherapy sensitivity or resistance [3, 4]. One of the most adverse features of, and difficulty in treating, these lesions is its high rate of recurrence demonstrated at 18.5% in a recent series, but noted as high as 45% in other reports [5]. While these lesions most commonly occur in the head and neck region, review of the otolaryngology literature shows a paucity of information regarding these uncommon tumors. We therefore present an interesting case of an MNTI presenting as a postauricular mass in a 2-month-old male and review the clinical, histopathologic, and radiographic features associated with these lesions.

## 2. Case Presentation

**Figure 1:**
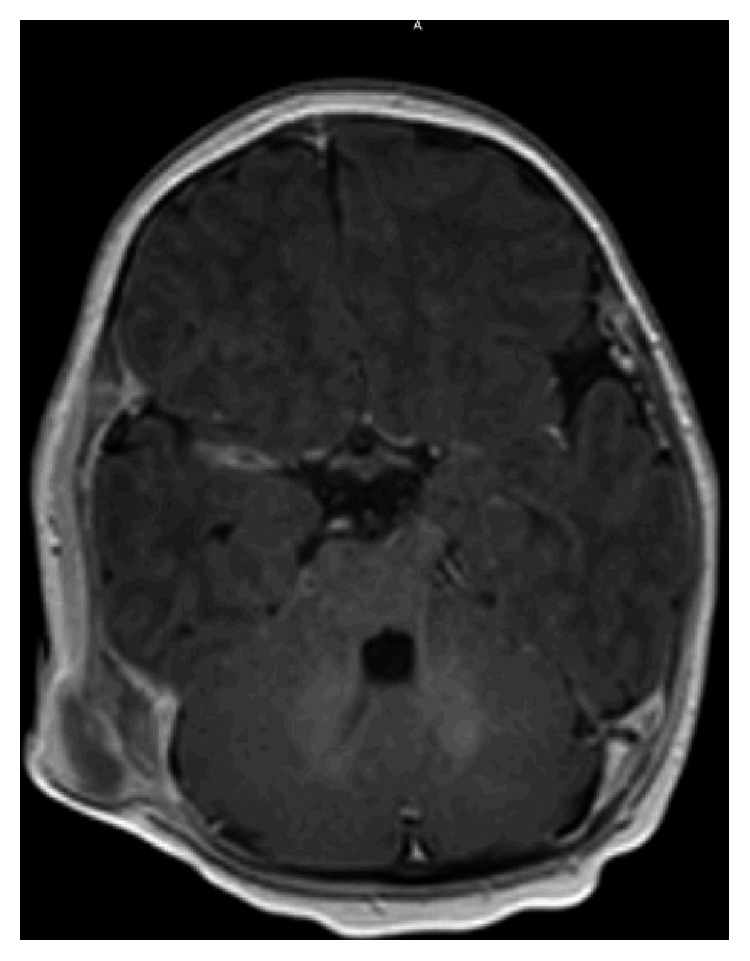
T1-contrasted sequence MRI of the initial lesion prior to any surgical therapy. Note: isointensity with signal abnormality of the adjacent temporal bone and temporoparietal suture line and peripheral enhancement representing areas of nonossification.

**Figure 2:**
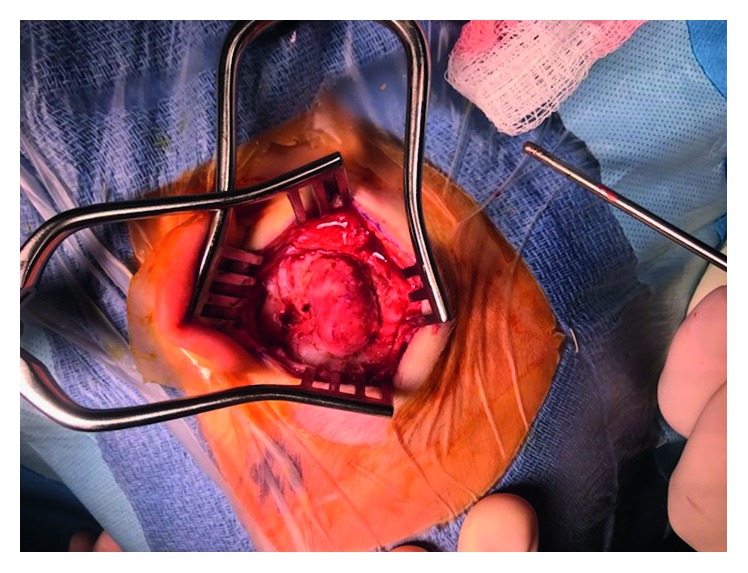
In vivo appearance of MNTI, demonstrating significant progression from 1.3 cm nodule on T1-contrasted sequence MRI two weeks prior. Note that a cortical mastoidectomy has been performed at the anterior (left) aspect to allow identification of the mastoid tegmen and landmarks for resection.

**Figure 3:**
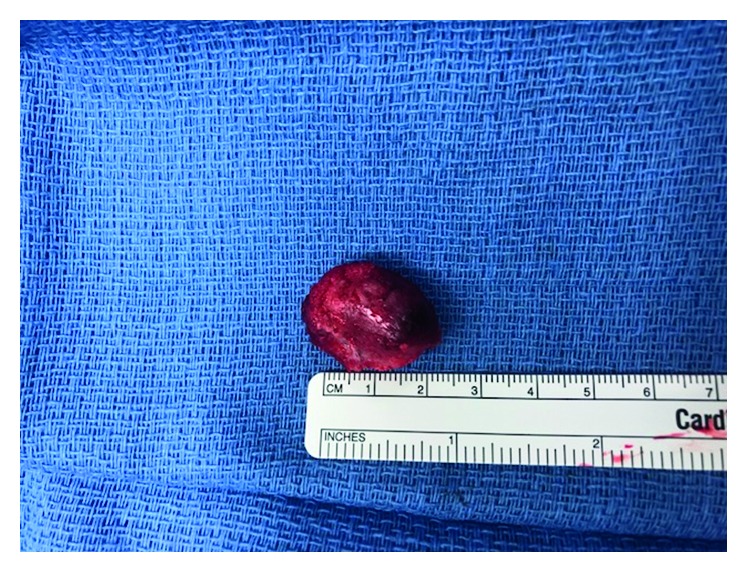
Ex vivo appearance of MNTI. Notice the dark blue characteristic and well-circumscribed nature of the lesion.

Our patient was a two-month-old male referred for evaluation of a left postauricular mass, present since birth. Workup by the patient's pediatrician including an ultrasound suggested a cystic mass prompting referral for surgical excision. The parents endorsed noticing the lesion at birth and that it had been painless and slowly progressive. Physical exam demonstrated a firm 2 × 2 cm subcutaneous lesion of the postauricular region. An MRI was obtained demonstrating a 2.3 × 1.4 × 2.2 cm well-defined solid mass involving the outer table of the right temporal bone and temporoparietal suture with intense peripheral enhancement and without restricted diffusion (Figure 1). Initial resection in the operating room was undertaken, and a deep plane between the mass and skull was identified and followed reflecting the lesion off of the skull. Unfortunately, pathology demonstrated focal presence of tumor cells at the peripheral margin. The patient underwent a repeat resection, with a canal wall up mastoidectomy. The lesion was again resected en bloc, and the underlying cortical bone was drilled down to the inner table of the temporal bone with healthy appearing bone stock. Despite clinically normal-appearing bone, the pathology again demonstrated presence of tumor cells at the soft tissue margins, and clinically the patient demonstrated significant regrowth of the lesion. The patient returned to the operating room once more, with a fairly impressive progression of gross tumor, nearly 2.5 × 2.0 cm (Figure 2). A revision mastoidectomy was performed, and neurosurgical consultation was obtained. The mass was excised en bloc resulting in a full-thickness craniectomy. The dura appeared healthy and unaffected by the tumor (Figure 3). The wound was closed primarily, and the patient was observed overnight in the PICU before being discharged home postoperative day one in stable condition. The patient developed purulence at his incision site one month postoperatively requiring intra-washout with neurosurgery. The infection resolved without further complication or treatment requirement. He was seen at six months postoperatively with no evidence of disease in good condition.

## 3. Discussion

**Figure 4:**
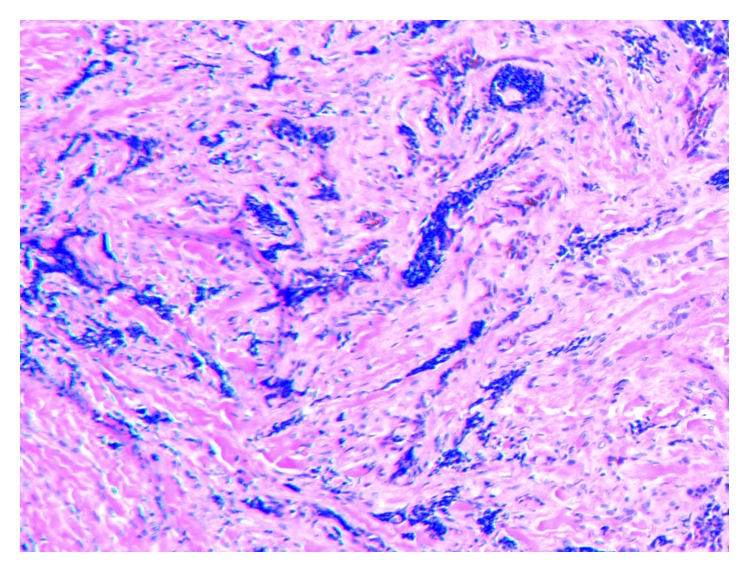
10x magnification of hematoxylin and eosin staining demonstrating spindle cells and small crushed blue cells.

**Figure 5:**
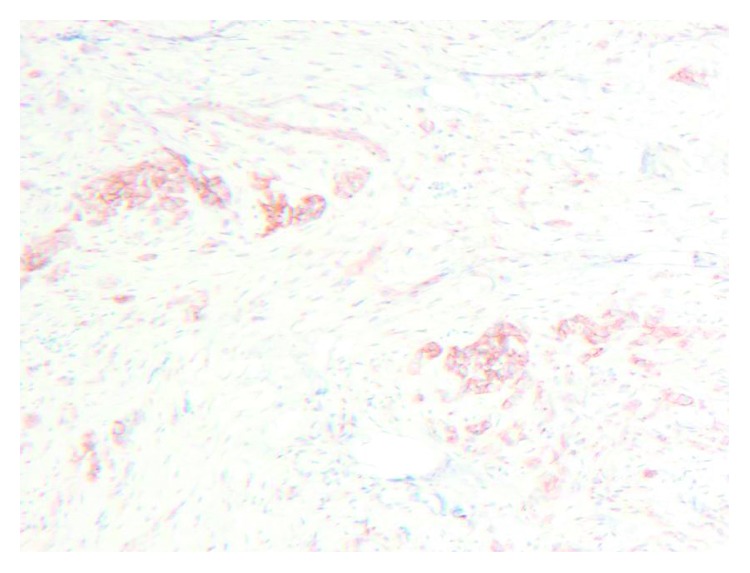
Immunohistochemical staining demonstrating CD99 positivity typically seen in the large melanin-containing cell population.

MNTI is a rare osteolytic tumor occurring in infants less than one-year-old and most frequently affecting the maxilla. The rarity of this entity makes anatomic prevalence difficult, but a 2015 systematic review of the literature suggested that less than one-fifth of these lesions occur in the nongnathic cranium. Another review in the neurosurgery literature suggests approximately five described cases occurring in the temporal bone [6]. These lesions are known to have a small but real malignant potential and are known to recur following surgical excision. Diagnosis is typically made on a histological basis, typically only after surgical excision. Common histological features of these tumors include the presence of two distinct cell populations: large epithelioid melanin-pigmented cells and small round “neuroblast-like” cells, lending to the tumors namesake. Immunohistochemical staining demonstrates cytokeratin, HMB45, and vimentin positivity in the larger epithelioid cells, and synaptophysin and glial fibrillary acidic protein (GFAP) positivity in the smaller round blue cells. Both of these cell types are usually negative for chromogranin A and neurofilaments. MNTI is usually negative for Ki-67 and CD99, but when present, it appears to be correlated with the more aggressive growth of the tumor [7]. The immunohistochemical staining characteristics for our patient is demonstrated in Table 1 and are consistent with the “classic” findings. Interestingly in our patient, CD99 was positive, possibly correlating with the recalcitrant nature of the mass and difficulty in clearing the lesion (Figures 4 and 5).

Review of the literature failed to demonstrate consistent, widely accepted radiographic findings; however, a recent publication suggested common MRI characteristics of these lesions with predominantly iso- or hypointense signal enhancement characteristic on T1- and T2-weighted images. Additionally, contrast enhancement on MRI is usually quite marked in all nonossified components of the tumor, which was again represented by our patient's lesion [8].

Standardized treatment consists of wide local excision; however, in our case, the ability to do this was hampered by critical neuro-otologic structures. Recurrence is common in these tumors, quoted at 20–45%. Our case reaffirms this finding as well as highlights the difficulty achieving negative resection margins despite the clinical margins appearing negative, making the ability to define the true extent of the lesion difficult.

## 4. Conclusion

Through coordinated effort, and sound surgical technique, MNTI can be treated successfully when recognized and addressed in a thoughtful manner. Our case demonstrates a unique otolaryngologic presentation of this rare tumor and the necessity, therefore, for the practicing pediatric otolaryngologist to be familiar with the characteristic and salient features of identifying and treating these lesions.

## Figures and Tables

**Table 1:** Tumor histochemical markers in our patient.

	Cytokeratin	HMB45	Vimentin	GFAP	CD45	NSE	Ki-67	CD99	S-100
Large melanin-containing cells	Positive	Positive	Positive	Negative	Negative	Positive	Negative	Positive	Negative
Small rounded neuroblast-like cells	Positive	Positive	Positive	Positive	Negative	Positive	Negative	Negative	Negative

## Abbreviations

MNTI: Melanocytic neuroectodermal tumor of infancy.
